# T cell responses to SARS-CoV-2 vaccination differ by disease-modifying therapy for multiple sclerosis

**DOI:** 10.1172/jci.insight.165111

**Published:** 2023-06-22

**Authors:** Asia-Sophia Wolf, Anthony Ravussin, Marton König, Mathias H. Øverås, Guri Solum, Ingrid Fadum Kjønstad, Adity Chopra, Trygve Holmøy, Hanne F. Harbo, Silje Watterdal Syversen, Kristin Kaasen Jørgensen, Einar August Høgestøl, Jon Torgils Vaage, Elisabeth G. Celius, Fridtjof Lund-Johansen, Ludvig A. Munthe, Gro Owren Nygaard, Siri Mjaaland

**Affiliations:** 1 Division of Infection Control, Section for Immunology, Norwegian Institute of Public Health, Oslo, Norway.; 2 Department of Neurology, Oslo University Hospital, Oslo, Norway.; 3 Institute of Clinical Medicine, University of Oslo, Oslo, Norway.; 4 Department of Immunology, Oslo University Hospital, Oslo, Norway.; 5 Department of Neurology, Akershus University Hospital, Lørenskog, Norway.; 6 Center for Treatment of Rheumatic and Musculoskeletal Diseases, Diakonhjemmet Hospital, Oslo, Norway.; 7 Department of Gastroenterology, Akershus University Hospital, Lørenskog, Norway.; 8 Department of Psychology and; 9 KG Jebsen Centre for B Cell Malignancy, University of Oslo, Oslo, Norway.

**Keywords:** COVID-19, Immunology, Cellular immune response, Multiple sclerosis, T cells

## Abstract

Immune responses in people with multiple sclerosis (pwMS) receiving disease-modifying therapies (DMTs) have been of significant interest throughout the COVID-19 pandemic. Lymphocyte-targeting immunotherapies, including anti-CD20 treatments and sphingosine-1-phosphate receptor (S1PR) modulators, attenuate Ab responses after vaccination. Evaluation of cellular responses after vaccination, therefore, is of particular importance in these populations. In this study, we used flow cytometry to analyze CD4 and CD8 T cell functional responses to SARS-CoV-2 spike peptides in healthy control study participants and pwMS receiving 5 different DMTs. Although pwMS receiving rituximab and fingolimod therapies had low Ab responses after both 2 and 3 vaccine doses, T cell responses in pwMS taking rituximab were preserved after a third vaccination, even when an additional dose of rituximab was administered between vaccine doses 2 and 3. PwMS taking fingolimod had low detectable T cell responses in peripheral blood. CD4 and CD8 T cell responses to SARS-CoV-2 variants of concern Delta and Omicron were lower than to the ancestral Wuhan-Hu-1 variant. Our results indicate the importance of assessing both cellular and humoral responses after vaccination and suggest that, even in the absence of robust Ab responses, vaccination can generate immune responses in pwMS.

## Introduction

Immune responses to SARS-CoV-2 in immunocompromised individuals have been of intense interest throughout the COVID-19 pandemic. In the absence of vaccination, immunologically vulnerable groups are especially susceptible to severe COVID-19 disease and hospitalization (reviewed in ref. 1); after vaccination, reduced responses to SARS-CoV-2 vaccines and potential vaccine failure have been of particular concern.

Multiple sclerosis (MS) is an immune-mediated disease characterized by inflammation and demyelination in the CNS. Current treatment involves modulation of the immune system to alleviate inflammation. However, some disease-modifying therapies (DMTs) can also impede an effective response to infectious diseases and vaccination (reviewed in ref. 2). It is unclear whether people with MS (pwMS) are more susceptible to severe COVID-19 disease in the absence of vaccination (3–6); current evidence suggests that this varies depending on DMT use, where treatment with anti-CD20 drugs presents an increased risk factor (7, 8), as well as neurological disability, comorbidities, and age (6, 8). It is, therefore, important to establish vaccine effectiveness in pwMS and whether vaccination protects against COVID-19 disease to the same extent as in the general population. Certain DMTs are known to be associated with increased risk of other infections: anti-CD20 drugs such as rituximab, ocrelizumab, and ofatumumab are associated with a range of serious infections, including respiratory tract infections (9, 10); sphingosine-1-phosphate receptor (S1PR) modulators, including fingolimod, ozanimod, and siponimod, which sequester lymphocytes in lymph nodes, are associated with increased risk of herpesvirus infections or reactivations (11); and natalizumab, an anti–α-4 integrin mAb, with a risk of progressive multifocal leukoencephalopathy (9).

The primary focus of many vaccine efficacy studies to date has been the humoral immune response (12, 13). Many DMTs, particularly anti-CD20 drugs, target B cells, and people receiving such treatments have reduced or nonexistent Ab responses after vaccination (14–18); pwMS taking fingolimod have been found to have significantly reduced Ab responses (14, 19). By contrast, pwMS taking other DMTs, including natalizumab (20, 21), cladribine (an adenosine mimic that triggers lymphocyte apoptosis) (22), and alemtuzumab (an anti–CD52 mAb that depletes T and B cells) (23) appear to have Ab responses comparable to those of untreated control groups.

Several studies have looked at cellular responses to SARS-CoV-2 spike peptides in pwMS after 2 doses of SARS-CoV-2 vaccine (16, 21, 24–26) and found that IFN-γ^+^ T cell responses were detectable in many, though not all, patients taking a variety of DMTs. One exception was pwMS treated with fingolimod (26), in whom T cell responses were significantly attenuated. Although not all individuals receiving anti-CD20 therapies developed T cell responses, it further appears that T cell responses and Ab titers are not well correlated, and so a lack of Ab response is not, in itself, indicative of a failed response to vaccination (27). Additionally, data on the effect of a third vaccine dose on both Ab levels and T cell responses are mixed; some studies suggest no effect of additional vaccination on either humoral or cellular immune responses (28), whereas others find boosted responses (29, 30).

The time between receiving a dose of DMT and vaccination varies between DMTs. Fingolimod, for example, is taken daily, whereas anti-CD20 treatments are administered at 6-month intervals, with a clear impact of this interval on the humoral response. An increased gap between administration of anti-CD20 therapies and vaccination is associated with stronger Ab responses (15, 27, 31–33), which may be beneficial during vaccination but can also lead to interruptions in ongoing treatment of MS or undesirable delay in vaccine schedules. It is, therefore, of interest to establish what effect ongoing DMT treatment has on vaccine responsiveness during both the primary vaccine course and for subsequent boosters.

Recent register studies indicate that pwMS treated with high-efficacy DMTs, including alemtuzumab, natalizumab, cladribine, S1PR modulators, and anti-CD20 therapies, have the best long-term outcomes for reduced worsening of disability and relapse outcomes (34, 35). Although, for safety reasons, alemtuzumab is rarely given to newly diagnosed patients, many people have been treated with this induction therapy during the past decade and comprise an important subset of pwMS. This study, therefore, focused on the cellular response to these 5 therapies that are among the most likely to be the treatments of choice for future pwMS.

The aim of this study was to investigate IgG Ab binding to the receptor binding domain (RBD) on the spike protein of SARS-CoV-2 as well as functional spike-specific CD4 and CD8 T cell responses of pwMS taking 5 different DMTs and of a healthy control group after 2 doses of SARS-CoV-2 vaccine. We also investigated whether a third vaccine dose improved the humoral and/or cellular responses in individuals treated with rituximab or fingolimod who had impaired IgG anti-spike RBD Ab responses after 2 vaccine doses.

## Results

A cohort of pwMS living in Oslo or Akershus, Norway, who were receiving treatment with DMTs prior to the beginning of the COVID-19 pandemic, were recruited as part of an ongoing population-based study of vaccine responses in pwMS in Norway (NevroVax) (15, 29). Cellular samples were collected from a subset of individuals taking different DMTs (namely, fingolimod, rituximab, cladribine, natalizumab, and alemtuzumab) both before and after the primary course of 2 vaccine doses between April and July 2021 (Supplemental Figure 1; supplemental material available online with this article; https://doi.org/10.1172/jci.insight.165111DS1). Healthy control participants were recruited from among health care workers at Diakonhjemmet Hospital and Akershus University Hospital. Rituximab- and fingolimod-treated individuals who had low Ab responses after vaccination were offered a third vaccine dose in the summer of 2021 (Trial registration EudraCT no. 2021-003618-37) before recommendations for booster vaccines in Norway were changed in September of that year to recommend a third dose for all immunocompromised individuals. The characteristics of this cohort are described in Table 1 according to DMT, including age, sex, time since last drug administration, and vaccine type (primarily the mRNA vaccines BNT162b2 [Comirnaty, Pfizer-BioNTech] and mRNA-1273 [Spikevax, Moderna]; see Methods for further details). The dosing schedule for rituximab was 1000 mg for the initial dose and 500 mg every 6 months thereafter. Fingolimod was taken daily. Breakthrough COVID-19 infections occurring more than 14 days after vaccination are shown for each group (*n* = 37 across all DMTs and healthy control participants). Infections were predominantly contracted between November 2021 and February 2022, representing a mixture of Delta and Omicron variants of concern (VOC) infections. None of the individuals were hospitalized or died.

**Figure 1:**
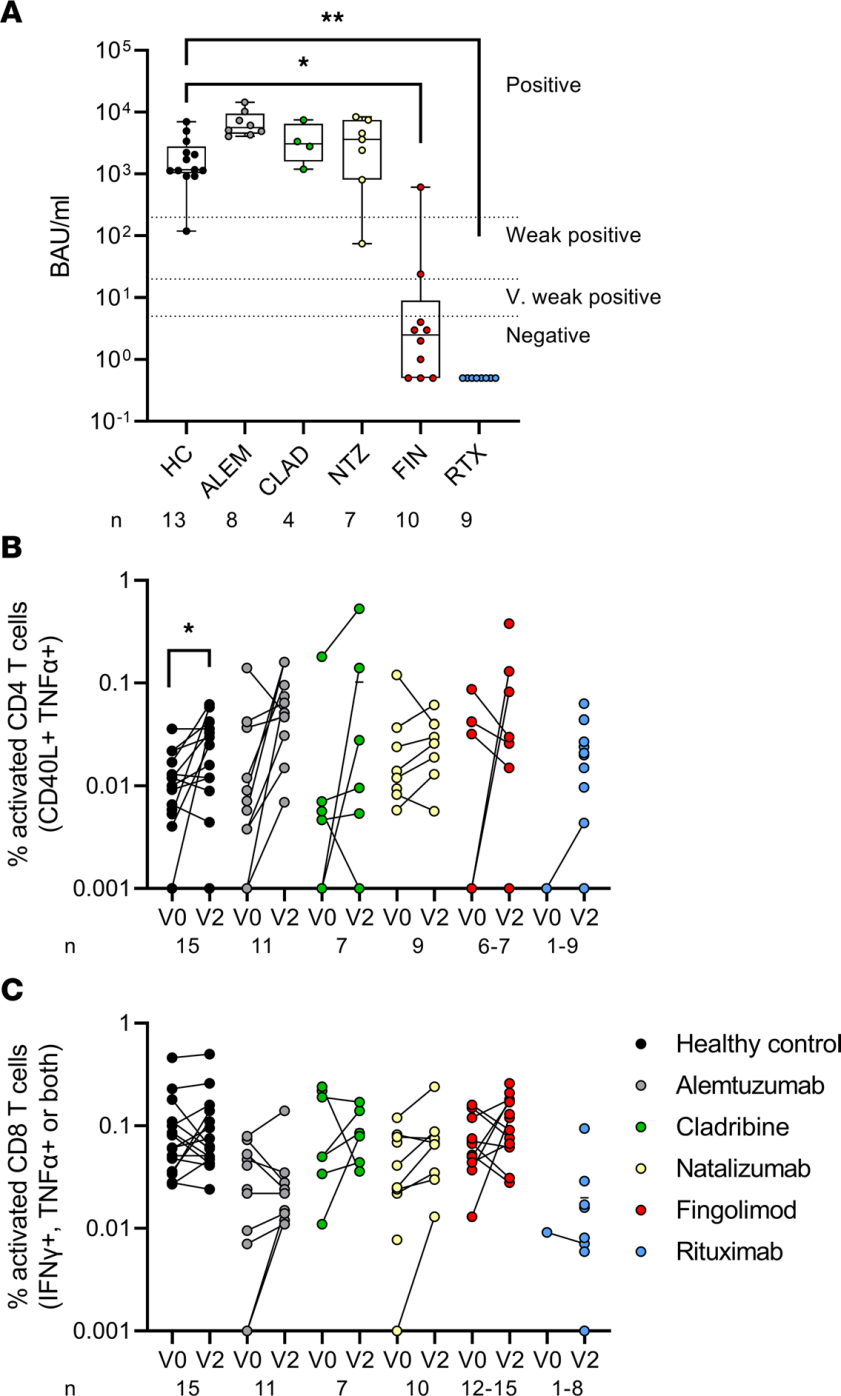
Responses after 2 vaccine doses in pwMS on DMTs. Individuals are grouped by DMT (healthy control participants [HC], and patients with MS who were treated with alemtuzumab [ALEM], cladribine [CLAD], natalizumab [NTZ], fingolimod [FIN], and rituximab [RTX]). (**A**) BAU values after 2 doses of vaccine. Responses below the lower limit of detection are shown as 0.5 BAU/mL; titers <5 BAU/mL are considered negative, 5–20 BAU/mL as very (V.) weak positives, 20–200 BAU/mL as weak positives, and >200 BAU/mL as positives. Individuals are shown as separate points; the box indicates median and IQR, whiskers indicate minimum and maximum values. Statistical analyses by Kruskal-Wallis test comparing DMT groups with HC with the Benjamini-Hochberg correction for multiple comparisons. (**B**) CD4 T cell (CD40L^+^ TNF-α^+^) and (**C**) CD8 T cell responses (IFN-γ^+^ and/or TNF-α^+^) to spike peptides before (V0) and after (V2) 2 doses of vaccine. Responses with zero events are plotted at 0.001% to indicate nonresponses. Samples from the same individual before and after vaccination are paired with a line. Statistical comparisons by Wilcoxon 2-tailed paired *t* tests. *P < 0.05, **P < 0.01, with the Holm-Šídák method for multiple comparisons. Patient numbers for each group are indicated along the *x* axis.

DMTs vary based on mechanism of action and cellular target. Therefore, we assessed the effect of each DMT on Ab levels and CD4 and CD8 T cell function by flow cytometry (Figure 1). The full flow cytometry gating strategy is shown in Supplemental Figure 2.

Samples taken 3 weeks (median 20.5 days) after the second vaccine dose were assayed for Ab binding activity (measured in binding Ab units [BAU]/mL) (Figure 1A). IgG anti-spike RBD responses were classified as negative (<5 BAU/mL), very weak positive (5–20 BAU/mL), weak positive (20–200 BAU/mL), and positive (>200 BAU/mL) and are indicated on the graph in Figure 1A for reference. All healthy control participants and individuals treated with alemtuzumab, cladribine, and natalizumab had strong Ab titers, predominantly in the positive range (median per group: healthy control participants, 1166 BAU/mL; alemtuzumab, 5591 BAU/mL; cladribine, 3081 BAU/mL; natalizumab, 3625 BAU/mL). However, individuals treated with fingolimod or rituximab had poor Ab responses after 2 vaccine doses (median: fingolimod, 2.5 BAU/mL; rituximab, 0.5 BAU/mL, which is below the level of detection for this assay).

T cell responses were assessed using activation-induced marker assays and measured by flow cytometry. PBMCs were stimulated with SARS-CoV-2 spike peptides and CD4 T cell activation was measured by CD40L and TNF-α coexpression (Figure 1B) before (V0; baseline) and 2 weeks after the second vaccination (V2). Samples were taken from the same individuals at both time points wherever possible, as indicated by paired dots in Figure 1B. There was a significant increase in the spike-specific CD4 T cell response after vaccination in the healthy control group (adjusted *P* = 0.045, Holm-Šídák correction for multiple Wilcoxon signed-rank tests). Likewise, the majority of patients treated with alemtuzumab had increased CD4 T cell responses after vaccination, suggesting that most of these individuals had reconstituted their immune system within the time since last treatment, which was more than 3 years for most patients. Responses were highly heterogeneous and did not reach statistical significance in the other DMT groups. More than half (*n* = 10 of 18) of the fingolimod-treated group had too few CD4 T cells in our assay to accurately measure activation responses and data from these participants were excluded from the analysis as there were too few CD4^+^ events to calculate the percentage response. CD8 T cell responses (Figure 1C) producing IFN-γ and TNF-α showed heterogeneity between individuals and generally increased after vaccination. Of note, as fingolimod prevents lymphocyte egress from lymphoid tissues (36), both flow cytometry profiles and whole blood cell counts showed significantly reduced numbers of circulating lymphocytes in fingolimod-treated pwMS compared with rituximab-treated pwMS (Supplemental Figure 3).

**Figure 2:**
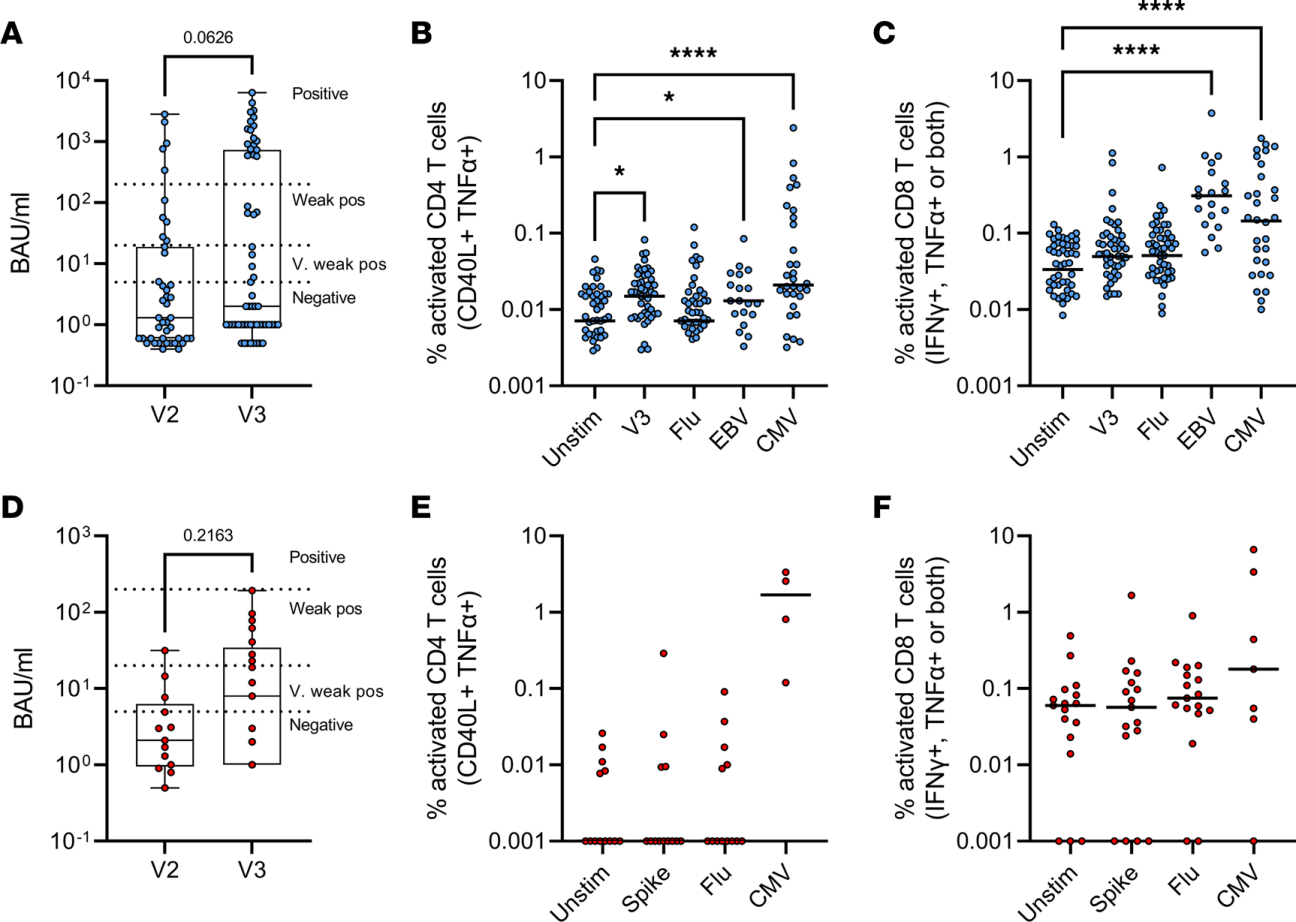
T cell and Ab responses in rituximab- and fingolimod-treated patients after third vaccine dose. Ab responses (BAU/mL) and CD4 and CD8 T cell responses after third vaccine dose in rituximab-treated (**A**–**C**) and fingolimod-treated patients (**D**–**F**) (**A**, *n* = 43–61; **B**, *n* = 21–56; **C**, *n* = 21–54; **D**, *n* = 13–21; **E**, *n* = 6–13; **F**, *n* = 6–17). Dotted lines in (**A**) and (**D**) indicate classification of Ab responses as negative or positive, as described previously; box-and-whisker plots indicate the minimum and maximum, median, and IQR. (**B**, **C**, **E**, and **F**) Lines on scatter plots indicate the median. Statistical analyses by 2-tailed Wilcoxon signed-rank tests with Benjamini-Hochberg FDR correction for multiple comparisons. *P < 0.05, ****P < 0.0001. Flu, influenza; Unstim, without stimulation; V. very.

As the fingolimod- and rituximab-treated individuals had poor Ab responses, these patients received a third dose of vaccine (see Methods) (Figure 2). Individuals treated with rituximab did not show significant improvements in IgG anti–spike RBD after a third vaccine dose (Figure 2A), although the overall responses and number of responders increased (median and IQR, respectively, at V2 were 1.3 and 18.3 BAU/mL; at V3, 2.0 and 665.5 BAU/mL; 13 of 43 individuals (30.2%) had >5 BAU/mL titers after 2 vaccine doses, increasing to 27 of 59 (45.7%) after 3 vaccine doses), suggesting that some, though not all, individuals had improved Ab responses after repeated vaccination. However, the number of patients who were Ab positive (>200 BAU/mL) was significantly increased from 5 of 43 after 2 doses to 19 of 59 after 3 doses (2-tailed *P* = 0.0185, Fisher’s exact test). Moreover, a significant proportion of individuals had detectable spike-specific CD4 and CD8 T cell responses (Figure 2, B and C), demonstrating that rituximab treatment does not inhibit T cell responses to the same extent as Ab responses.

The same effect was not seen for people treated with fingolimod. After a third vaccine dose, fingolimod-treated patients showed no significant increase in IgG anti–spike RBD (Figure 2D) and, generally, had even lower Ab responses than the rituximab-treated group, with no patients reaching the positive response classification of >200 BAU/mL (median and IQR at V2, 2.1 and 5.35 BAU/mL; at V3, 8.0 and 33.5 BAU/mL). Despite an increase in weak responders (20–200 BAU/mL), this was not statistically significant (1 of 13 individuals had weak responses after V2 compared with 7 of 20 after V3; 2-tailed *P* = 0.108, Fisher’s exact test). Spike-specific CD4 and CD8 responses also showed no significant response, suggesting that fingolimod has a major impact on measurable T cell responses in blood as well as on Ab levels.

A majority (61.4%; *n* = 51 of 83) of fingolimod- or rituximab-treated patients received an influenza vaccine between September 2020 and February 2021. However, influenza-specific T cell responses did not significantly differ between individuals who had received a seasonal influenza vaccination during the previous winter (2020/21) and those who had not. This suggested that T cell responses generated via previous vaccination or influenza infections prior to the COVID-19 pandemic were still detectable in these patients. There was a weak but significant positive correlation between CD4 responses to SARS-CoV-2 spike and CD4 responses to influenza peptides in rituximab-treated patients, suggesting individual differences to vaccine antigens in general (Supplemental Figure 4A). T cell responses to EBV and CMV peptides were higher than responses to the vaccine peptides, which represent the difference between vaccination and latent viral infection. In rituximab-treated individuals, we saw strong CMV-specific CD4 and CD8 T cell responses (both *P* < 0.0001, Wilcoxon signed-rank tests) (Figure 2B) and EBV-specific CD4 and CD8 T cell responses (*P* = 0.013 and *P* < 0.0001, respectively) (Figure 2C). However, there was no correlation between CD4 T cell responses to spike and CMV in rituximab-treated patients (Supplemental Figure 4B), CD4 and CD8 T cell responses to spike (Supplemental Figure 4C), or CD4 responses and Ab responses (Supplemental Figure 4D), consistent with other studies showing low concordance between these measures of immune responsiveness (27).

**Figure 3:**
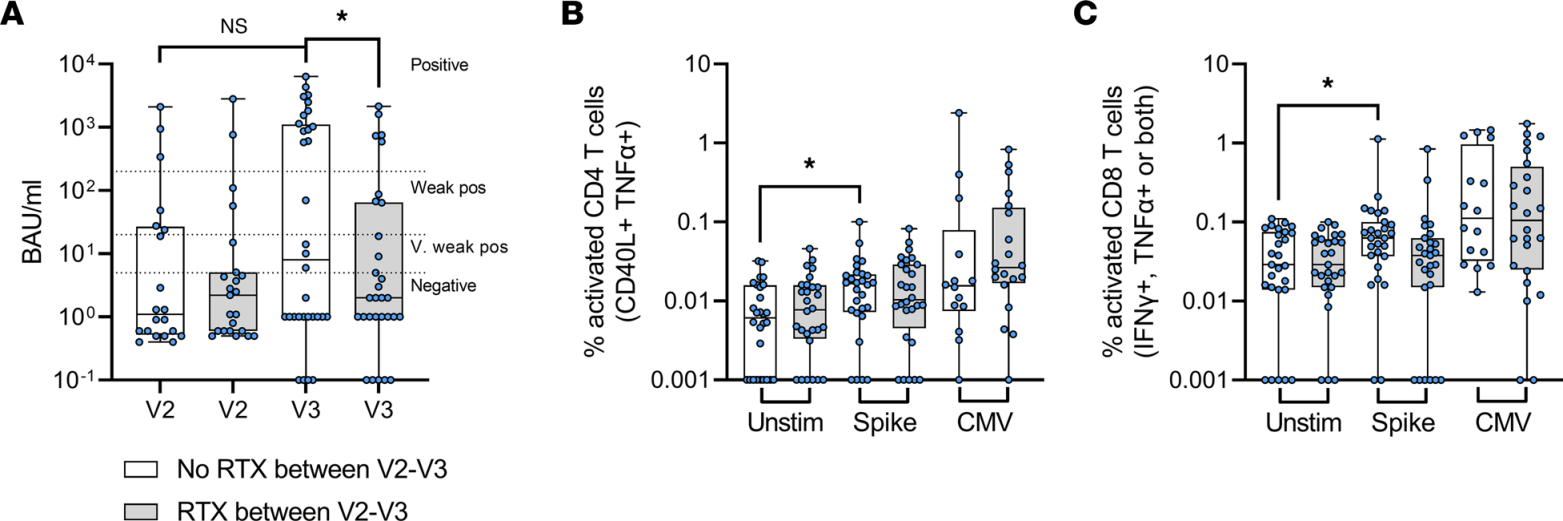
Readministration of rituximab (RTX) between vaccine doses affects Ab but not T cell responses. Rituximab-treated individuals were grouped by whether they received a dose of RTX between V2 and V3. (**A**) Ab titers (BAU/mL) after the V2 and V3 vaccine doses for patients who did not receive RTX between vaccines (empty boxes) (*n* = 20–32) and patients who did receive RTX between vaccines (gray boxes) (*n* = 23–30). (**B**) CD4 T cell and (**C**) CD8 T cell responses without stimulation (unstim) or to SARS-CoV-2 spike or CMV peptides after third vaccine dose for patients without (*n* = 28) or with RTX administration (*n* = 28) between vaccine doses. Statistical analyses for paired responses by Wilcoxon signed-rank tests, unpaired responses by Mann-Whitney tests, and 2-tailed *P* values are shown, *P < 0.05. All *t* tests were corrected for multiple comparisons by Benjamini-Hochberg FDR method.

The administration interval of DMTs varies by drug, as described in Table 1. In the course of this study, patients taking rituximab received treatment according to their individual schedules. All patients received rituximab prior to the baseline (V0) sample and completed the initial 2-dose vaccine course without additional rituximab infusions. Between the second and third vaccine doses, approximately half of the patients (*n* = 30 of 62) received another dose of rituximab (median time 8.43 weeks before V3; range, 1.86–19.7 weeks). We hypothesized that this rituximab dosage impaired the ability to respond to vaccination. Ab and T cell responses in these 2 groups, therefore, were compared (Figure 3).

There was no significant difference in IgG anti–spike RBD between these 2 groups after V2, but after V3, individuals who had recently received rituximab had significantly lower Ab activity (Figure 3A) than those who had not (*P* = 0.023, unpaired *t* test). However, there was no such difference between the T cell responses of the 2 groups (Figure 3, B and C). Comparing the spike-specific responses between groups showed no difference in CD4 (*P* = 0.874, Mann-Whitney test) or CD8 T cell activation (*P* = 0.033, not significant with Benjamini-Hochberg correction for FDR), suggesting that T cell responses are not affected by readministration of rituximab after the primary vaccine course.

**Figure 4:**
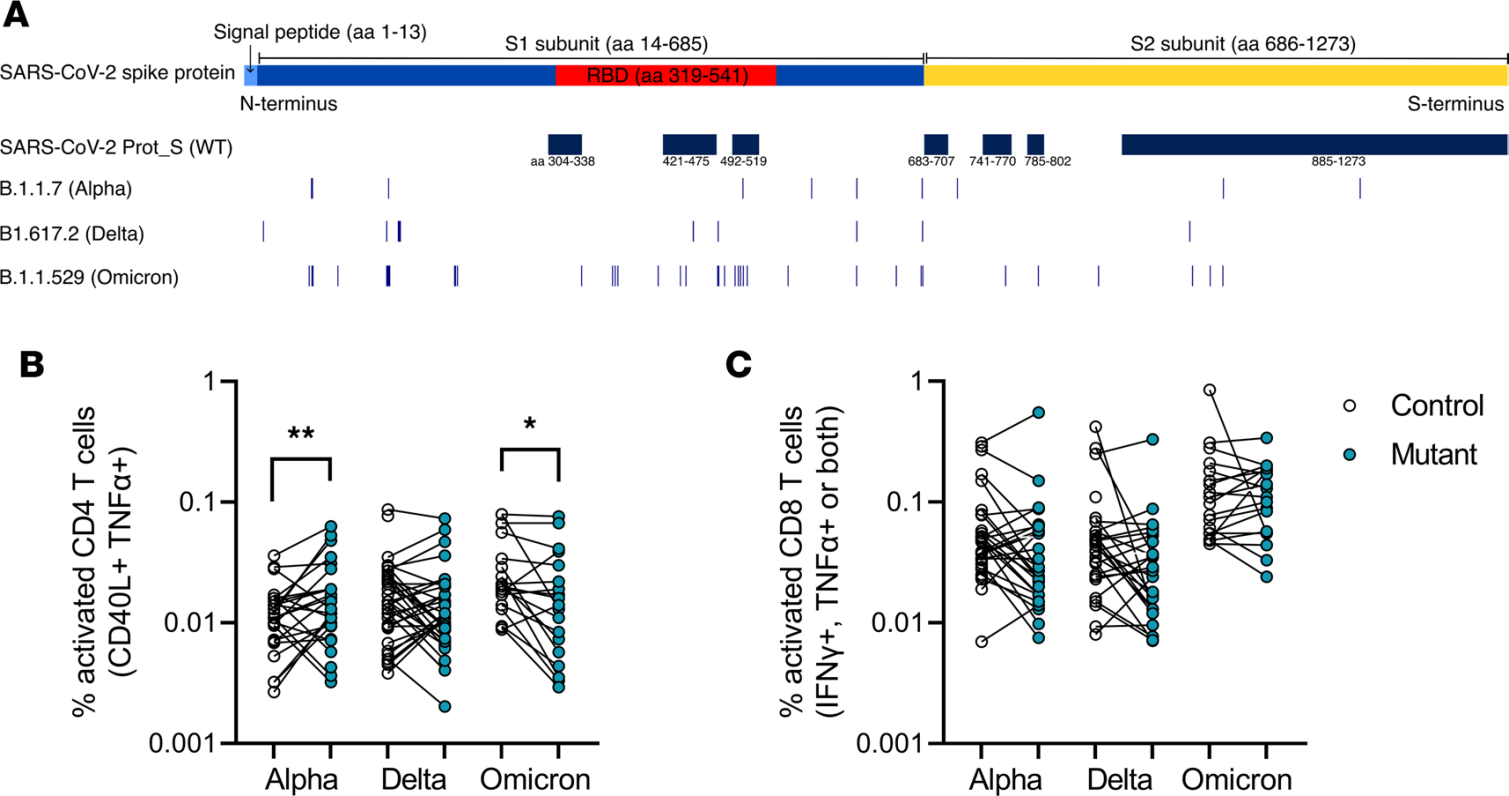
T cell responses to the Delta and Omicron SARS-CoV-2 variants after 3 vaccine doses. (**A**) Schematic of mutated regions in the Alpha, Delta, and Omicron regions stimulated by peptides. The SARS-CoV-2 spike protein is 1273 aa long and consists of the signal peptide and S1 and S2 subunits; the RBD in S1 is indicated in red (59). Regions covered by the SARS-CoV-2 Prot_S (WT) peptide used for activation-induced marker assays are shown for reference. The control and mutant peptides for each variant cover the same loci but with the mutated (mutant) or Wuhan-Hu-1 variant (control). The aa mutations are listed in Methods. PBMCs from rituximab-treated patients after a third vaccine dose were stimulated with spike peptide pools from the mutated regions (blue circles) of the Alpha (*n* = 29), Delta (*n* = 41), and Omicron (*n* = 21) VOC and the same regions of the WT sequence (empty circles), and the CD4^+^ (**B**) and CD8^+^ (**C**) T cell responses were compared. Statistical differences were calculated by Wilcoxon signed-rank tests with the Holm-Šídák method for multiple comparisons. *P < 0.05, **P < 0.01.

The question of whether vaccination confers protection against SARS-CoV-2 VOC has been of particular concern since the initial emergence of the Alpha (B.1.1.7) variant and subsequent Delta (B.1.617.2) and Omicron (B.1.1.529/BA.1-5) variants. Mutations in the spike region of these variants are thought to reduce the ability of vaccine-generated Abs to recognize these variants and potentially to reduce protection against them. To measure how T cell responses from vaccination were affected, we assessed CD4 and CD8 T cell responses to the mutated peptides of these 3 variants (Figure 4). PBMCs from triple-vaccinated, rituximab-treated patients were stimulated as before with only the mutated peptide regions from the Alpha, Delta, or Omicron variants, as well as the homologous peptides for each variant from the original Wuhan-Hu-1 sequence. The location and number of mutated peptides (34, 32, and 83 peptides for Alpha, Delta, and Omicron, respectively) are shown in Figure 4A. CD4 T cell responses to the Alpha variant increased slightly compared with the homologous Wuhan-Hu-1 sequence (adjusted *P* = 0.009, Wilcoxon signed-rank test with Holm-Šídák correction) but were significantly reduced against the mutated peptides of the Omicron variant (adjusted *P* =0.018) (Figure 4B). Although CD8 T cell responses were reduced, particularly for the Delta VOC, these differences did not reach significance (Figure 4C).

**Figure 5:**
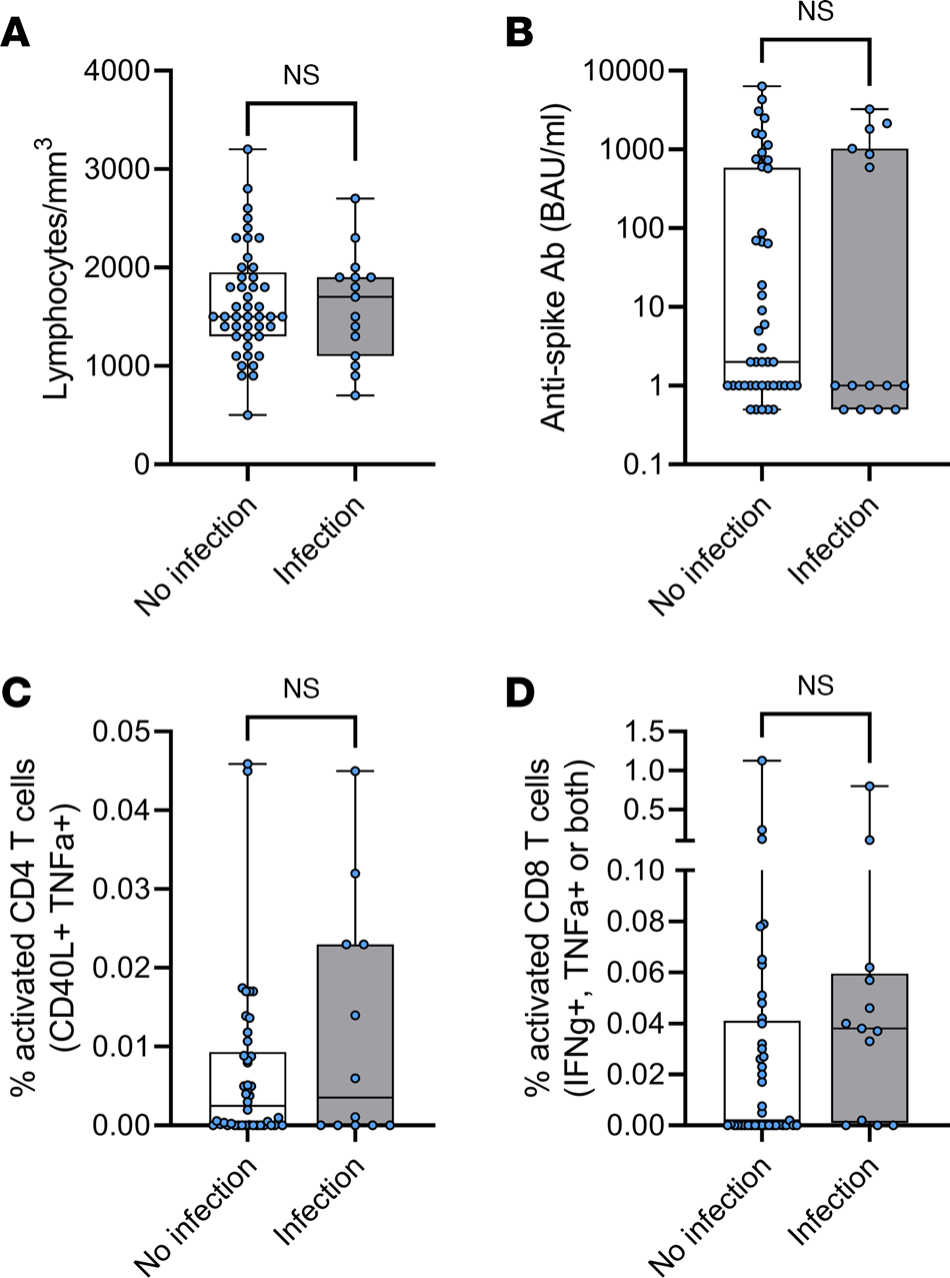
Immune responses after 3 vaccine doses do not predict subsequent infection. PwMS taking rituximab who were subsequently infected with SARS-CoV-2 up to February 2022 (gray boxes; *n* = 15) did not have significantly different lymphocyte counts (**A**), BAU levels (**B**), activated CD4 T cells (**C**), or activated CD8 T cells (**D**), compared with pwMS taking rituximab who were not infected in the same time period (white boxes; *n* = 45). Boxes represent the median and IQR, whiskers represent the minimum and maximum values; all individuals shown as separate points. (**C** and **D**) T cell responses show frequencies of spike-specific responses with unstimulated background subtracted. Statistical differences were calculated by Mann-Whitney unpaired *t* tests, All results were NS.

Of the rituximab- or fingolimod-treated pwMS in this study, 17 were subsequently infected with SARS-CoV-2 between July 2021 and February 2022 after 3 vaccine doses, at least 14 days after vaccination. None of these individuals required hospital treatment or oxygen supplementation, and none died. Of the rituximab-treated individuals with subsequent infections (*n* = 15), there were no significant differences in lymphocyte counts, Ab levels, and CD4 or CD8 T cell responses after vaccination but before infection, compared with people who were not infected in the time period considered here (*n* = 45) (Figure 5). The time between last vaccine dose (V3) and infection (in days; median 141, range 17–221) did not correlate with Ab levels or T cell responses (data not shown). All individuals received a third vaccine in either early July (*n* = 16) or late August 2021 (*n* = 44); of the people who were subsequently infected between July 2021 and February 2022, 6 had been vaccinated in the earlier group (37.5%) and 9 in the later group (20.5%) (*P* = 0.195, Fisher’s exact test). The mean time between vaccination and infection for each group was 161 and 122 days, respectively. Just over half of individuals (*n* = 7 of 15) had received rituximab between the second and third vaccine doses, but this did not have a statistically significant effect on the likelihood of subsequent infection with SARS-CoV-2 (compared with 22 of 45 of pwMS who were not infected with SARS-CoV-2 in this time frame; *P* > 0.999, Fisher’s exact test). Viral sequencing data were not available but were likely to be a mix of the Delta and Omicron strains.

## Discussion

Older and immunocompromised individuals are particularly at risk of severe COVID-19 disease. Vaccine efficacy in immunocompromised individuals is, therefore, important to understand, particularly as many countries, including Norway (37), have achieved high vaccine coverage and have since lifted many or all infection-limiting measures such as social distancing. However, SARS-CoV-2 variants continue to circulate and vulnerable groups may still be at risk of severe disease. These data show that pwMS treated with alemtuzumab, cladribine, and natalizumab have robust humoral and CD4 and CD8 T cell responses after 2 vaccine doses, in agreement with other studies (3, 14, 20, 22, 25), although it should be noted that alemtuzumab-treated individuals had last received treatment an average of 3 years previously, which may have contributed to higher Ab responses comparable to individuals without MS; although T cell populations are suppressed for up to 5 years after treatment, B cell populations recover within months (38).

However, individuals treated with fingolimod and rituximab had strongly reduced Ab responses compared with both healthy control participants and pwMS taking other DMTs. Upon receipt of a third vaccine dose, both treatment groups had small increases in IgG anti-spike levels, and a significantly increased percentage of patients in the rituximab-treated group developed high responses (>200 BAU/mL), demonstrating that some individuals were capable of increasing B cell responses. This finding was also found in a larger study in which improved IgG anti-spike responses were found after third vaccination (29). Moreover, triple-vaccinated, rituximab-treated individuals demonstrated both CD4 and CD8 T cell responses against SARS-CoV-2 spike peptides. These T cell responses were not reduced even when individuals received rituximab between their second and third vaccine doses, suggesting that although readministration of anti-CD20 drugs does impair humoral responses, cellular responses are preserved.

Additionally, we observed strong CD4 and CD8 T cell responses to the herpesviruses CMV and EBV in pwMS, suggesting that specific T cell responses against antigens from long-term latent infections are present. T cell activation against other vaccine antigens such as influenza were comparable to the SARS-CoV-2 spike-specific responses, and rituximab-treated individuals showed a positive correlation between spike-specific and flu-specific responses. This suggests that vaccine responsiveness varies by individual but is not necessarily associated with T cell responses to other infections such as CMV. Immune responses may also differ based on whether individuals were already using DMTs at the time of antigen exposure, which may affect the magnitude of the immune response, as well as the availability and duration of antigen seen during vaccination or acute infection compared with chronic infections.

In pwMS treated with fingolimod, a third vaccine dose did not appear to improve either the Ab or T cell responses. Because fingolimod is taken daily, the fluctuations in B cell counts seen in individuals taking anti-CD20 drugs are not seen (39). The reduction in CD4 T cell percentages we observed here was consistent in individuals at different sampling points, suggesting that the administration of fingolimod causes lymphocyte sequestration to different extents for each individual. This observation is consistent with other studies that have shown that S1PR modulators change the profile of peripheral blood immune subsets and particularly retain CD4 T cells, compared with CD8 T cells in lymphoid tissue (reviewed in ref. 40), as well causing an overall decrease in the number of circulating lymphocytes (36, 40). Although Ab titers were strongly reduced for all fingolimod-treated patients, we observed that individuals with less skewed CD4 to CD8 T cell ratios had stronger spike-specific CD8 T cell responses, suggesting that people with higher circulating CD4 T cell frequencies were more likely to generate measurable and potentially protective cellular responses. Similarly, a clinical trial found that Ab responses after 3 vaccine doses correlated strongly with lymphocyte counts in pwMS who had been taking fingolimod prior to the trial (41).

Nevertheless, pwMS receiving fingolimod do not appear to be at higher risk of severe COVID-19 or hospitalization than the general population prior to vaccination (7, 42). Fingolimod has been found to reduce proinflammatory cytokine release from DCs and monocytes (43) which may reduce detrimental uncontrolled inflammation associated with severe COVID-19 disease (44). Additionally, as lymphocytes are sequestered rather than destroyed by S1PR modulators (36, 45), failure to detect T cell responses in peripheral blood may not fully reflect the extent of the total T cell response, and noncirculating cellular responses induced by vaccination may be present in the lymph nodes or other secondary lymphoid organs.

Several large-scale studies have found that pwMS taking fingolimod or ocrelizumab are at higher risk of SARS-CoV-2 infection after vaccination than the general population or pwMS taking other DMTs (46–48), possibly reflecting the role of circulating Abs in preventing infection. However, the severity of these infections is still unclear, and where cases could be followed on an individual level, there were no deaths from COVID-19 (47, 48). Further research has found that, even after vaccination, pwMS taking anti-CD20 drugs were at higher risk of hospitalization but not death; this risk was not seen with other DMTs, including S1PR modulators (49). In our study, 32 individuals across all DMTs contracted SARS-CoV-2 after vaccination and none of these were hospitalized or died. There were no significant differences in Ab or T cell responses, after three vaccine doses, in the 15 rituximab-treated pwMS who were infected with SARS-CoV-2 during the study time frame compared with other people taking rituximab; there was also no significant effect of time since last vaccine on the date of infection. There may be an effect of time since the last treatment with rituximab, as this has a clear effect on Ab levels, but we did not find a statistically significant difference in likelihood of infection between people in our cohort who had recently received rituximab and those who had not. These data are complicated by the emergence and rapid dominance of the Omicron VOC in Norway and Europe over this time (late 2021–early 2022) and are likely to be affected by the prevalence of circulating SARS-CoV-2 infections in the general population.

The emergence of SARS-CoV-2 VOC has further complicated the question of vaccine efficacy and protection. Neutralizing Abs against the Delta and Omicron VOC have been found to be sharply reduced compared with the original Wuhan-Hu-1 strain (50), whereas T cell responses are more heterogeneous and show wide cross-reactivity to other human coronaviruses as well as between variants (51–53). In triple-vaccinated, rituximab-treated individuals, we found that CD4 T cell responses to the Omicron VOC were reduced compared with WT, suggesting that although T cells are responsive to the mutated VOC regions, vaccine-generated T cell–mediated protection may be reduced. However, these mutated regions cover only a fraction of the spike peptide sequences, and further work is needed to determine how these mutations affect T cell vaccine responses.

We found no correlation between any combinations of Ab titer, CD4 T cell responses, or CD8 T cell responses; therefore, using only 1 of these parameters as an indication of immune responsiveness cannot give a full picture of vaccine efficacy. Although the correlates of protection against SARS-CoV-2 infection and severe COVID-19 disease are still unclear and the relative roles of Ab-mediated virus neutralization and T cell–dependent protection are still being extensively studied (54–56), analysis of cellular responses in addition to Ab titers can give a better understanding of whether immunosuppressed individuals are likely to require additional protective measures. Further follow-up studies are required to determine whether T cell responses in the absence of Ab titers, such as seen in our rituximab-treated population, are protective against severe disease, but the current evidence supports the contention that T cell immunity is sufficient.

One limitation of this work is a lack of longitudinal sampling to measure changes in CD4 and CD8 T cell responses between the second and third vaccine for the rituximab- and fingolimod-treated patients. The question of whether repeated vaccination with antigens from the Wuhan-Hu-1 variant can prevent disease from successive VOC remains to be determined, as does the impact of the newer Omicron VOC–targeting vaccines on generating variant-specific T cell responses.

In summary, we found that pwMS taking DMTs that inhibit Ab responses can still mount T cell responses comparable with that of healthy control participants and, furthermore, that continued administration of the widely used anti-CD20 drug rituximab between the primary vaccine course and subsequent vaccine doses does not impede cellular responses. Further analyses of the efficacy and durability of cellular responses, and well as the impact of additional vaccination, are needed to better understand how vaccines protect against severe disease in immunocompromised individuals.

## Methods

### Participant recruitment and ethical approvals.

All patients from the Norwegian MS registry (*n* = 12,000) in 2021 were invited to participate in the humoral arm of the NevroVax study, which was designed to investigate vaccine responses in individuals with MS who were receiving immunomodulating therapies (EudraCT registration no. 2021-003618-37). A subgroup of patients from Oslo University Hospital taking the DMTs alemtuzumab, cladribine, natalizumab, fingolimod, and rituximab (approximately 10 patients per DMT) were recruited to provide PBMC samples, along with all patients who lacked Ab responses after 2 vaccine doses (considered at the time to be <70 AU/mL by ELISA). Individuals from Oslo University Hospital, Akershus University Hospital, and Haukeland University Hospital who had low humoral responses subsequently received a third vaccine dose, and those treated at Oslo University Hospital or Akershus University Hospital comprised the fingolimod- and rituximab-treated individuals at V3. Healthy controls were recruited among health care workers from Diakonhjemmet Hospital and Akershus University Hospital, and samples were stored in the Oslo University Hospital biobank.

### Vaccination and inclusion in vaccination trial.

PwMS were vaccinated per guidelines of the Norwegian Corona Vaccination Program, where immunocompromised individuals and health care workers (who participated in this study as in the healthy control group) were the high-priority groups. Vaccines were administered according to the manufacturers’ recommendations and health administration advice at the time, ranging from 3 weeks between first and second doses for mRNA-1273 and 6 and 10 weeks for BNT162b2. Some individuals received first doses of ChAdOx1-S (Vaxzevria, AstraZeneca), the distribution of which was subsequently discontinued in Norway in March 2021, and second doses of BNT162b2. Individuals who had a COVID-19 infection before or during the course of vaccination were excluded from further analyses. Individuals who did not seroconvert to IgG anti-spike (RBD) after the standard 2 doses were invited to participate in a vaccination trial to receive a third dose of mRNA-1273 or BNT162b2 outside the framework of the Norwegian Corona Vaccination Program (EudraCT registration no. 2021-003618-37). Patients included in this study after September 1, 2021, received third-dose vaccines following revised guidelines in the Norwegian Corona Vaccination Program (whereby all immunocompromised adults were advised to receive a third dose).

### Sample collection.

Venous blood for PBMC isolation was collected at Oslo University Hospital into BD Vacutainer CPT tubes with sodium citrate. Tubes were centrifuged for 20 minutes at 1600*g* to isolate PBMCs, which were then pipetted into fresh tubes, washed twice with RPMI, and frozen in 90% FCS (Gibco, Thermo Fisher) with 10% DMSO (Sigma Aldrich) in liquid nitrogen for future use.

### T cell stimulation and flow cytometry.

Cryopreserved PBMCs were thawed in RPMI, washed thrice to remove residual DMSO, and counted. Cells were plated into 96-well, U-bottomed plates at 200,000 cells per well and stimulated for 24 hours in RPMI culture medium containing 10% FCS, 1 mM sodium pyruvate (Gibco, Thermo Fisher), 1x MEM NEAA (Gibco), 50 nM 1-thioglycerol, and 12 μg/mL gensumycin. GolgiPlug (BD Biosciences) containing brefeldin A was added after 2 hours of stimulation until the end of the incubation. Cells were stimulated with the following peptide pools: PepTivator SARS-CoV-2 Prot_S, covering the immunodominant sequence domains of the spike glycoprotein from the SARS-CoV-2 (Wuhan-Hu-1 variant); EBV consensus; and CMV pp65 pool (used according to the manufacturer’s recommendations at 0.75nmol/mL; all Miltenyi Biotec); and pooled pan-influenza peptides for HLA class I and II (final concentration 1 μg/mL) (GenScript). Peptide pools for mutated SARS CoV-2 spike are outlined in the next paragraph. Cytostim (Miltenyi Biotec) was used as a positive control according to the manufacturer’s recommendation.

After 24 hours, cells were centrifuged at 500*g* for 5 minutes, the supernatant discarded, and cells resuspended in FACS buffer (1% FCS in PBS). Cells were centrifuged again and the supernatant removed. Cells were incubated with 10 μL surface Ab cocktail (anti–human CD3-BV605 (clone SK7; BD Biosciences); and CD4-eFluor 450 (OKT-4), CD8-AF488 (OKT-8), and Fixable Live/Dead Near-IR (1:1000 dilution) (all Thermo Fisher) for 30 minutes at 4°C, washed in FACS buffer, then fixed in Fix/Perm (BD Biosciences) for 20 minutes at room temperature. Cells were then washed twice with PermWash (BD Biosciences) and incubated with 10 μL intracellular Ab cocktail [anti–human IFN-γ–BV711 (clone 4S.B3), CD40L-BV510 (24–31) (both BioLegend); TNF-α-PE (Mab11) and CD69-APC (FN50) (both BD Biosciences)] for 30 minutes at room temperature. Cells were finally washed with PermWash and resuspended in 200 μL FACS buffer for analysis by flow cytometry within 24 hours.

Cells were acquired on a Bio-Rad ZE5 flow cytometer and analyzed with FlowJo software, version 10.7 (BD Life Sciences).

### VOCs and mutated peptide sequences.

Three PepTivator SARS-CoV-2 VOC spike protein Mutation Pools and the 3 corresponding spike protein WT Reference Pools (all Miltenyi Biotec) were used at a final concentration of 0.75 nmol/mL per the manufacturer’s recommendation. Prot_S B.1.1.7 Mutation Pool (catalog 130-127-844) included 34 peptides from 10 mutations: deletion 69, deletion 70, deletion 144, N501Y, A570D, D614G, P681H, T716I, S982A, and D1118H. The corresponding nonmutated peptide pool control was Prot_S B.1.1.7 WT Reference Pool (catalog 130-127-841).

Prot_S B.1.617.2 Mutation Pool (catalog 130-128-763) included 32 peptides from 10 mutations: T19R, G142D, E156G, deletion 157, deletion 158, L452R, T478K, D614G, P681R, and D950N. This subvariant lacks the E484Q mutation. The nonmutated peptide pool control was Prot_S B.1.617.2 WT Reference Pool (catalog 130-128-761).

Prot_S B.1.1.529/BA.1 Mutation Pool (catalog 130-129-928) included 83 peptides from 37 mutations: A67V, H69 deletion, V70 deletion, T95I, G142D, V143 deletion, Y144 deletion, Y145 deletion, N211 deletion, L212I, insertion 214EPE, G339D, S371L, S373P, S375F, K417N, N440K, G446S, S477N, T478K, E484A, Q493R, G496S, Q498R, N501Y, Y505H, T547K, D614G, H655Y, N679K, P681H, N764K, D796Y, N856K, Q954H, N969K, and L981F. The nonmutated peptide pool control was Prot_S B.1.1.529/BA.1 WT Reference Pool (catalog 130-129-927).

### Ab quantification.

Semiquantitative measurement of Abs to full-length spike protein (Spike-FL) and the RBD from SARS-CoV-2 was performed using a multiplexed bead-based assay as described in (57). Polymer beads with fluorescent barcodes were coupled to successively to neutravidin (Thermo Fisher) and biotinylated viral antigens to generate bead-based protein arrays. Sera were diluted 1:100 in assay buffer (PBS, 1% Tween-20, 10 μg/mL d-biotin, 10 μg/mL neutravidin, 0.1% sodium azide). Diluted serum samples were incubated with bead-based arrays in 384-well plates for 30 min at 22^o^C at constant agitation, washed 3 times in PBS/1% Tween-20, and labeled with R-phycoerythrin (R-PE)–conjugated goat anti–human IgG (Jackson ImmunoResearch). For measurement of neutralizing Abs, the beads were pelleted after incubation with serum and labeled successively with digoxigenin-conjugated human ACE2 and mouse monoclonal anti-dixogigenin (Jackson ImmunoResearch), which was conjugated in-house to R-PE. The beads were analyzed with an AttuneNxT flow cytometer (Thermo Fisher), and raw data (fcs.3.1) were analyzed in WinList 3D (Verity Softwarehouse). The R-PE median fluorescence intensity (MFI) of each bead subset was exported to Excel. The MFI of beads coupled with viral antigens was divided by that measured on beads coupled with neutravidin only (relative MFI [rMFI]). A total of 979 prepandemic serum samples and 810 serum samples from individuals convalescing from COVID-19 were analyzed to establish cutoffs for seropositivity. A double cutoff of rMFI of greater than 5 for anti-RBD and anti–Spike-FL yielded a specificity of 99.7% and a sensitivity of 95% (58). Serum from an individual who had received 3 doses of the Pfizer/BioNTech anti–COVID-19 vaccine was used as standard to convert signals to BAU/mL.

### Statistics and analysis.

Statistical analyses were performed using GraphPad Prism, version 9, for Windows (GraphPad Software). All *t* tests were 2-tailed. A *P* value of less than 0.05 was considered significant. We corrected *t* tests (Wilcoxon signed-rank tests for paired analyses or Mann-Whitney tests for unpaired analyses) for multiple comparisons using the Benjamini-Hochberg FDR with an FDR of 0.05. Adjusted *P* values are specified in the text and were corrected with the Holm-Šídák method. For analysis of functional markers (CD40L^+^ TNF-α^+^ CD4 T cells, and IFN-γ^+^ TNF-α^+^ CD8 T cells), data from FACS plots with fewer than 1000 CD4 or CD8 T cells were excluded.

### Study approval.

The study was approved by the Norwegian South-Eastern Regional Ethical Committee (Reference numbers 200631, 235424, 135924, and 204104), and the Norwegian Medicines Agency (EudraCT 2021-003618-37). All participants gave written informed consent prior to inclusion in this study.

## Author contributions

LAM and SM conceived and designed the study. ASW, AR, MK, LAM, TH, GON, and SM contributed to drafting the manuscript. ASW, AR, GS, IFK, and AC performed experiments. ASW, AR, MK, GS, IFK, FLJ, GON, LAM, and SM analyzed and interpreted data. MHØ, AC, TH, HFH, EAH, JTV, and EGC contributed to data analysis and interpretation. MK, GON, MHØ, SWS, KKJ, JTV, FLJ, and LAM organized the collection of samples and information from the cohorts. ASW, AR, and MK are joint first authors with the order determined by contribution to experimental analysis and writing of the manuscript. All authors contributed to and approved the final manuscript.

## Supplementary Material

Supplemental data

Supporting data values

## Figures and Tables

**Table 1:**
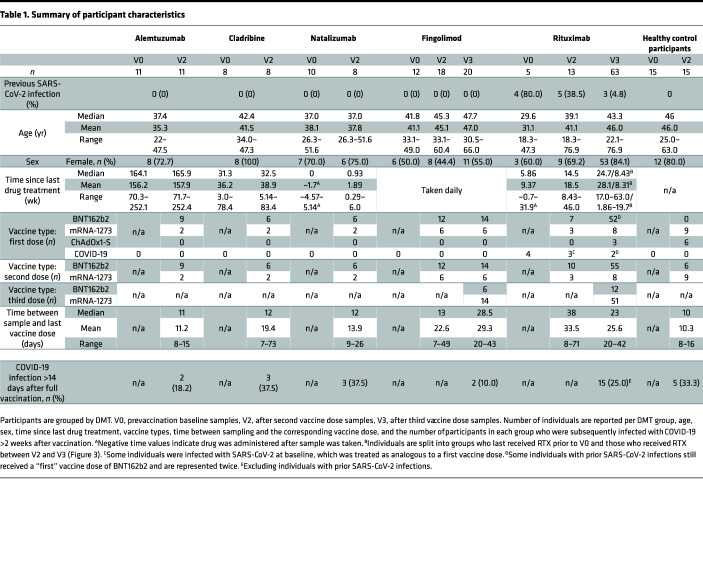
Summary of participant characteristics

## Version 1 05/09/2023

In-Press Preview

## Version 2 06/22/2023

Electronic publication
